# Nondestructive Determination of Diastase Activity of Honey Based on Visible and Near-Infrared Spectroscopy

**DOI:** 10.3390/molecules24071244

**Published:** 2019-03-29

**Authors:** Zhenxiong Huang, Lang Liu, Guojian Li, Hong Li, Dapeng Ye, Xiaoli Li

**Affiliations:** 1 College of Mechanical and Electrical Engineering, Fujian Agriculture and Forestry University, Fuzhou 350002, China; 1161213001@fafu.edu.cn (Z.H.); 1181218002@fafu.edu.cn (L.L.); 1181213003@fafu.edu.cn (G.L.); 3171218003@fafu.edu.cn (H.L.); 2 Fujian Engineering Research Center for Modern Agricultural Equipment, Fujian Agriculture and Forestry University, Fuzhou 350002, China; 3 College of Biosystems Engineering and Food Science, Zhejiang University, Hangzhou 310058, China

**Keywords:** visible and near-infrared spectroscopy, honey, kinetic mechanism, diastase activity, diastase number, spectral pretreatment methods, least squares-support vector machine

## Abstract

The activities of enzymes are the basis of evaluating the quality of honey. Beekeepers usually use concentrators to process natural honey into concentrated honey by concentrating it under high temperatures. Active enzymes are very sensitive to high temperatures and will lose their activity when they exceed a certain temperature. The objective of this work is to study the kinetic mechanism of the temperature effect on diastase activity and to develop a nondestructive approach for quick determination of the diastase activity of honey through a heating process based on visible and near-infrared (Vis/NIR) spectroscopy. A total of 110 samples, including three species of botanical origin, were used for this study. To explore the kinetic mechanism of diastase activity under high temperatures, the honey of three kinds of botanical origins were processed with thermal treatment to obtain a variety of diastase activity. Diastase activity represented with diastase number (DN) was measured according to the national standard method. The results showed that the diastase activity decreased with the increase of temperature and heating time, and the sensitivity of acacia and longan to temperature was higher than linen. The optimum temperature for production and processing is 60 °C. Unsupervised clustering analysis was adopted to detect spectral characteristics of these honeys, indicating that different botanical origins of honeys can be distinguished in principal component spaces. Partial least squares (PLS) and least squares-support vector machine (LS-SVM) algorithms were applied to develop quantitative relationships between Vis/NIR spectroscopy and diastase activity. The best result was obtained through Gaussian filter smoothing-standard normal variate (GF-SNV) pretreatment and the LS-SVM model, known as GF-SNV-LS-SVM, with a determination coefficient (*R*^2^) of prediction of 0.8872, and root mean square error (RMSE) of prediction of 0.2129. The overall results of this paper showed that the diastase activity of honey can be determined quickly and non-destructively with Vis/NIR spectral methods, which can be used to detect DN in the process of honey production and processing, and to maximize the nutrient content of honey.

## 1. Introduction

During the last several years, honey consumption has increased because it is a natural product composed of sugars, enzymes, amino acids, organic acids, carotenoids, vitamins, minerals, and aromatic substances [1,2]. The composition, color, aroma, and flavor of honey depend mainly on the flowers and geographical regions involved in its production, and are also affected by processing, manipulation, packaging, and storage time [3,4,5]. Honey-making is a very complicated process which takes more than 7 days from nectar to full maturity, and stores in the hive and covered with beeswax. Getting ripe honey involves using a knife to cut off the honey cover, which is very laborious and affects the production when it is brewed by bees. Therefore, many bee farmers usually do not wait until the honey is fully ripe to get the honey, and then use the concentrator to process it into concentrated honey through the heating process. There are more than 180 substances in honey, but it so distinctive and helpful is primarily due to the presence of enzymes, which were brought by bees during the nectar processing [6]. Activated enzymes are very sensitive to high temperature and will lose their activity when they exceed a certain temperature. However, concentrated honey will go through high temperatures in the process of concentration, leading to inactivation of a large number of active substances. Therefore, it is necessary to explore the effects of different heating conditions on the activity of enzymes in honey.

Honey contains multiple enzymes at low-concentrations, the most prominent of which are diastase, invertase (a-glucosidase), glucose-oxidase, catalase, and acid phosphatase [7]. As one of the most important enzymes, diastase (α- and β-amylase) not only enriches the nutritional and therapeutic function of honey, but it is also taken as an important index to evaluate honey qualities. The diastase activity is usually expressed in Schade units [8], also known as the diastase number (DN), which is defined as the amount of enzyme that will convert 0.01 g of starch to the prescribed end-point in 1 h at 40 °C under the conditions of the test. According to the Honey Quality and International Regulatory Standards, the diastase activity must not be less than or equal to 8, determined after processing and blending for all retail honey, and the activity must not be less than 3 for honeys with naturally low enzyme content. However, the traditional wet chemical method for the determination of DN is complicated and time-consuming, which does not meet the requirements of real-time and rapid and monitoring of enzyme activity in the honey heat treatment of the concentration. There is an urgent demand for a nondestructive and rapid method to measure DN.

In recent years, spectroscopy has become a hot technique in food detection applications. High-performance liquid chromatography typically takes a long time to complete and is destructive to the samples; most Fourier transform infrared spectroscopy systems require stable operating conditions, which limits the applicability of this method, particularly outdoors [9]. Neither of the two methods can meet the requirement of nondestructive and fast honey processing. The near infrared (NIR) technique has expanded its scope of application in the past decade, and self-renewal has been achieved through rapid integration of technologies from other fields. A portable, low-cost spectrophotometer, which communicates through wireless technology, has become a reality. NIR is a democratizing technique based on these tiny instruments when needed, applying them to non-professionals [10]. Near-infrared spectroscopy is a speedy, simple, and non-destructive method that requires no chemical reagents and allows several analytes to be detected simultaneously [11]. This method has been widely used in the field of honey quality detection, such as with respect to 5-hydroxymethyl furfural [12], variety discrimination [13], honey adulteration [14,15], moisture, and reducing sugar content [16]. Nevertheless, to date, attempts to attain fast and nondestructive prediction of diastase activity have not been successful.

The heating process represents one of the crucial steps during the honeys commercial processing, which can dissolve undesirable crystallization, reduce the moisture content, eliminate the microorganisms responsible for fermentation and spoilage, and affect the activity of diastase. In order to better guide the production, improve the quality and efficiency of honey processing, and protect the interests of consumers, the research contents of this paper are as follows: 1) to explore the kinetic mechanism of raw honey from different sources under different heating conditions; and 2) rapid and nondestructive detection of diastase activity was realized by using visible and near-infrared (Vis/NIR) spectroscopy techniques.

## 2. Results

### 2.1. DN Variation of Different Heat Treatments and Different Botanical Origins

**Figure 1:**
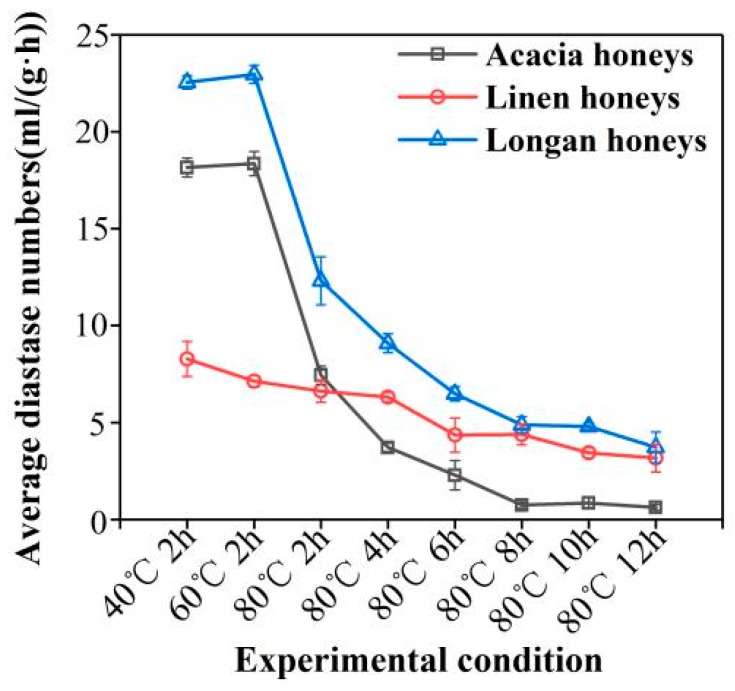
DN of the heat-treated honeys. Changes of diastase number in acacia, linen and longan honeys with different heating conditions.

DN results of the heat-treated honeys are shown in Figure 1. As can be seen from Figure 1, the overall diastase activity ranged from 0.56–23.11 in the tested heat-treated honeys (details are given in Table S1) 

For the heat-treated group (Figure 1), 5 parallel experiments were launched to confirm DN, and an averaged value was calculated as the final value of each group. The diastase activity decreased with the increase of temperature and heating time. Honey with a higher initial DN (18.30 and 22.63 DN units of acacia and longan) was heated, and the diastase activity exhibited a sharp decline in the first 2 h at 80 °C. The acacia and linen honeys are very sensitive to temperature. This is followed by a slow DN diminution period, which drops approximately 3 DN units per two heating hours until the sixth hour at 80 °C, at which point DN does not change further due to the severe deficiency of diastase. Bathing at 40 °C and 60 °C for 2 h exerted a minor effect on the diastase activity of honey, and therefore, 60 °C is the best processing temperature for honey. On the other hand, for honey with low initial DN (7.29 DN units, unknown heat-treated linen honeys), diastase activity was slowly diminishing by one DN unit per 2 h in the first 6 h of heating under 80 °C, and one DN units per 4 h from the sixth hour to the twelfth. Linen honeys had a comparatively low initial DN and were not as sensitive to heating as the other two honeys. The observed pattern of the DN decline confirmed by the previous research [2,17].

### 2.2. Spectral Characteristics

**Figure 2:**
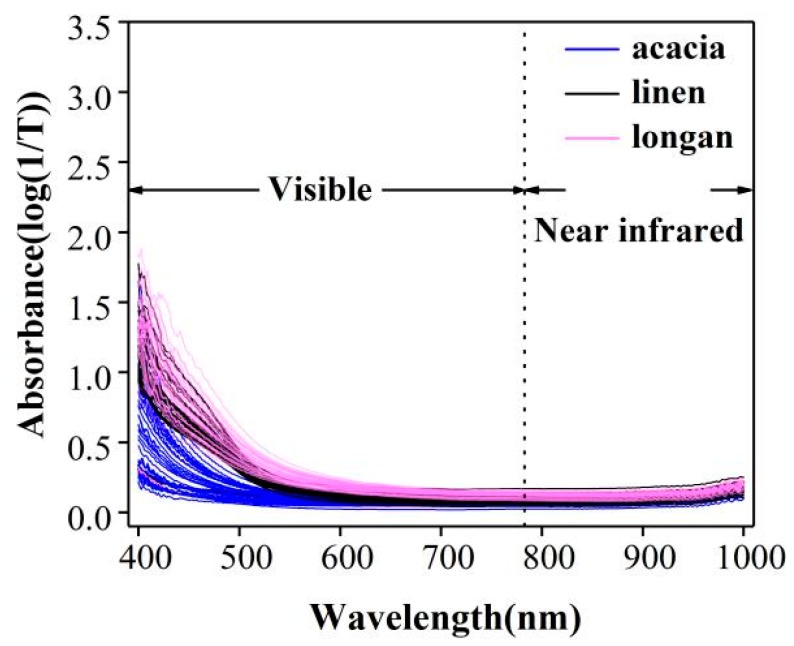
Visible (400–780 nm) and near-infrared (780–1000 nm) spectra of all the 110 honeys samples. Vis/NIR spectra of acacia (*n* = 37), linen (*n* = 35) and longan (*n* = 38) honeys.

The spectrum of all samples within the spectral range of 400–1000 nm was shown in Figure 2 (Details are given in Table S2). Electromagnetic spectra of both visible and shortwave near-infrared spectroscopy were analyzed. As can be seen in Figure 2, especially 400–700 nm, the spectra of different origins were quite informative and varied from each other. Even though the honey is from the same biological origin, their spectra possesses disaffinity due to the different heating conditions. Therefore, the different DN in honeys can be classified according to Vis/NIR spectroscopy data.

### 2.3. Spectral Cluster Analysis of Honey from Botanical Origins

**Figure 3:**
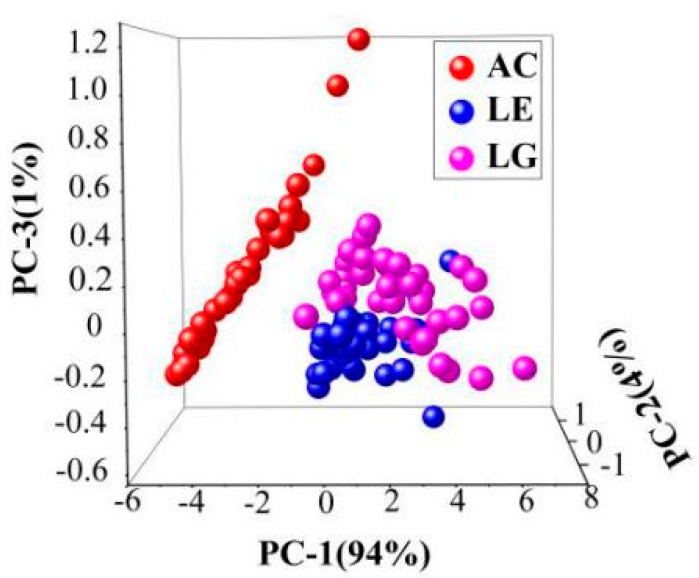
Cluster analysis of origins of honey from three botanical origins. (AC, LE, and LG represent acacia, linen, and longan separately). Acacia honeys can be clearly distinguished from linen and longan honeys, and the results of the cluster between linen and longan honeys are closed.

Honey from different biological sources could hardly be directly seen in the Vis-NIR spectra in Figure 2. To establish a simple classification model of honey from different origins, 110 samples (37 acacia, 35 linen, 38 longan) were investigated, and the range of 400–1000 nm was selected for principal component analysis (PCA). The results are shown in Figure 3, in which the first three PCs accounted for 99% of the total variance in the set of honey samples analyzed. The acacia can be clearly distinguished from linen and longan, and the results of the cluster between linen and longan are closed. However, except for a few abnormal samples, linen and longan showed better aggregation and could be separated. It is known that heating has an enormous influence on the samples, and SPA has better classification effects on different biological sources of honeys.

### 2.4. Establishment of the DN Determination Model

In order to realize fast nondestructive detection of diastase activity in processing, the quantitative relationship between the Vis/NIR spectroscopy and the activity of honey enzymes was to be established.

#### 2.4.1. Comparison of Different Pretreatment Methods

It is recognized that the proper use of chemometric methods, including pre-processing and calibration, is of vital importance to a successful prediction work. Insufficient use cannot lead to preferable results; however, too much will eliminate the useful information in the original data. It is significant for modeling purposes to find the appropriate pretreatment method. Several pretreatment methods were used in this study: Savitzky-Golay (SG) smoothing, Savitzky-Golay smoothing-standard normal variate (SG-SNV), Gaussian filter smoothing-standard normal variate (GF-SNV), and multiplicative scatter correction (MSC). After the pre-processing of data, samples were separated into two sets, calibration, and prediction, in the approximate ratio of 2:1 for each DN level. Both linear (PLS) and non-linear (LS-SVM) calibration methods were applied to the data, which established a quantitative regression model for spectrum and diastase activity, and the performance relationships between PLS and LS-SVM were found within these data. The results of PLS and LS-SVM models based on these pretreatment methods were used to assess the performances of these pretreatments and were shown in Table 1.

Comparing with PLS and LS-SVM models, it was found that LS-SVM models have a higher value of the determination coefficient (*R*^2^) in calibration and prediction and is more suitable for predicting diastase activity. As for LS-SVM models, all pretreatments improved the performances, and GF-SNV was the most effective with the highest *R*^2^ of prediction. By GF-SNV-LS-SVM models, the *R*^2^ of calibration was improved from 0.9772 to 0.9785, and the *R*^2^ of prediction was improved from 0.6017 to 0.8872; Meanwhiles, the GF-SNV-LS-SVM models have lower values of the root mean square error (RMSE). In synthetical consideration of the developed models, the original spectral data modeled by the GF-SNV-LS-SVM models have the optimal results for predicting the DN in honeys.

#### 2.4.2. Selection of the Characteristic Wavelengths

**Figure 4:**
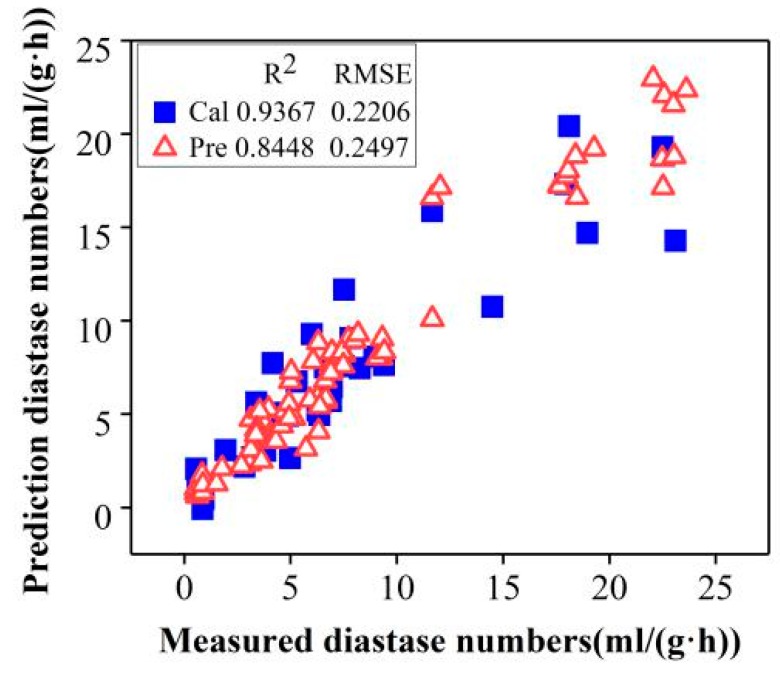
Measured vs. predicted diastase numbers in honeys by GF-SNV-LS-SVM models based on the characteristic wavelengths (Cal and Pre represent calibration and prediction, separately).

Building based on the full wavelength range of 400–1000 nm, containing 401 wavelengths, GF-SNV-PLS models should be further simplified to reduce the complexity of the model and the computing time. Therefore, SPA was utilized to select the characteristic wavelengths to reduce the complexity of the Vis-NIR spectroscopy data. After the operation of selecting the optimal wavelengths, the GF-SNV-LS-SVM models were built to assess the performance of predicting diastase activity in honeys. The results were shown in Figure 4.

**Figure 5:**
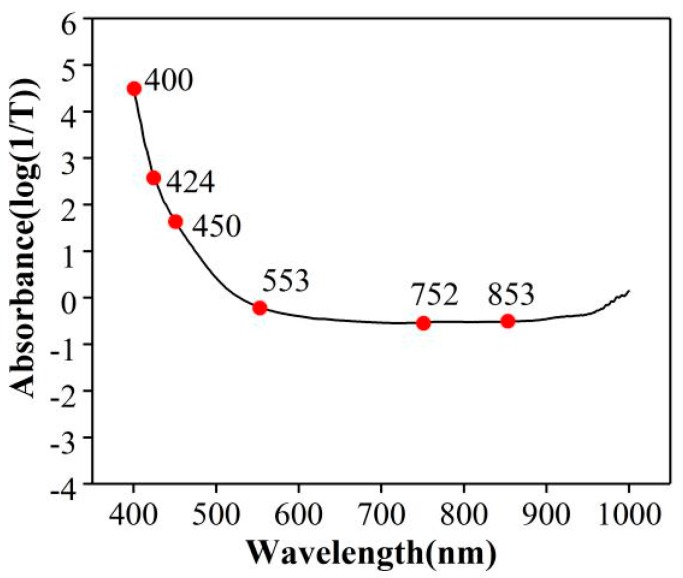
Characteristic wavelengths of the spectra based on the pretreatment of GF-SNV. SPA selected 6 characteristic wavelengths in red dot.

SPA selected 6 characteristic wavelengths for LS-SVM models after the pretreatment of GF-SNV, among which most of the characteristic bands were located between 400–700 nm, and the results were shown in Figure 5. In the VIS region, the area around 425 nm shows the highest loadings and contributes to the discrimination of the floral origin [18]. The functional group of O-H (6ν) belongs to alkyl alcohol around 553 nm and may be related to volatile oils constituents, O-H (4ν) belongs to hydrocarbon around 752 nm, and C-H (4ν) belongs to aromatics around 853 nm related to aromatic esters in honeys [19].

#### 2.4.3. Establishment of Non-Linear Determination Models

Generally, better performances have been found in LS-SVM models with different pre-processing methods compared with PLSR models. From Table 1, it can be seen that the performance of the LS-SVM regression model is superior to the PLS regression model from beginning to end. As seen in Figure 4, the wavelengths were reduced from 401 to 6 by SPA, which greatly improved the efficiency of the detection. The influence of wavelength decrease on model precision was assessed by building GF-SNV-LS-SVM models based on these characteristic wavelengths. The results of GF-SNV-LS-SVM models were shown in Figure 5.

The GF-SNV-LS-SVM models based on the characteristic wavelengths obtained comparable results with the GF-SNV-LS-SVM models based on the full spectral range with the determination coefficient of prediction of 0.8448 and 0.8857, separately. It demonstrated that the characteristic wavelength selection not only greatly simplified the model imports, but also remained the steadiness and the fitting degree of the models. The results showed that GF-SNV-LS-SVM was the optimization model for rapid and nondestructive prediction of diastase activity.

This result represents the only and best outcome in the prediction of DN using NIR technique, and the result indicated important meanings for further study in two aspects. On the one hand, this result demonstrated the potential of evaluating diastase activity in a fast and non-destructive way. On the other hand, the generally superior performances compared with PLS demonstrate that LS-SVM is a powerful tool for spectral analysis.

## 3. Discussion

In this paper, the kinetic mechanism of the effect of temperature on the activity of diastase in honey was studied, and a nondestructive method for rapid determination of diastase activity in honey was developed. First of all, thermal treatment was adopted as the means to acquire the different levels of diastase activity of original honeys and explore the dynamic change of diastase activity under heating treatment. The results showed that the time of deactivation of diastase activity varies from species to species, and heating at 80 °C for more than 4 h will destroy the diastase efficacy of acacia and longan honeys. Heating at 80 °C was a severe heating treatment to honeys, especially for the long duration of hours, but there was still some active diastase existing even after 6 h of heating. This phenomenon further confirmed the fact that honey diastase was heat-resistant. In order to slow down the natural crystallization process and ensure its stability during commercial life, raw honey is usually pasteurized before packaging to dissolve sugar crystals and destroy yeast [20]. Previous studies have shown that heating at 80 °C for 15 min reduced diastase activity [8], and 60 °C is the recommended temperature for honey production and processing, which is lower than the temperature used for pasteurization of honey [21]. The results of this paper strongly indicated a conclusion that the time of deactivation of diastase activity varies from species to species, and heating at 80 °C for more than 4 h will destroy the diastase efficacy in the honeys [22].

Secondly, diastase content in the honey varies from species to species. In the study of Reference [23], it can be proven that diastase activity is closely related to biological sources. Thirdly, quantitative determination models of enzyme activity are developed based on Vis/NIR spectroscopy with high prediction accuracy, indicating that Vis/NIR spectroscopy can be used for the nondestructive and rapid detection of enzyme activity. Furthermore, the fingerprint wavelengths were detected based on the characteristic wavelength selection algorithm, and their assignment indicated that this Vis/NIR spectral determination has a stable chemical basis. Honey from different biological sources has diverse colors, which has proved in the literature that honey sources are distinguished based on colors in the Vis-NIR region using PCA [18,21,24]. In the visible region, especially at 400–425 nm, a clear absorption peak can be attributed to the color variation of honeys [18]. Fourthly, in this study, the characteristic bands selected by SPA indicated that the vibration and stretching of functional groups of substances in honey can be detected using spectroscopy. The functional groups of O-H (6ν) at around 553 nm, O-H(4ν) at around 752 nm, and C-H (4ν) at around 853 nm may belong to volatile oils constituents, hydrocarbon, and aromatic esters in honeys, separately [20]. Vis/NIR spectroscopy can detect the vibration and stretching of O-H (6ν), O-H (4ν), and C-H (4ν) to predict DN in honeys.

Finally, it has been found that there are some connections between the performances of PLS and LS-SVM models based on different pre-processing methods for this set of data, and because of that, both of these two calibration methods highlight the useful factors in the processed data for DN analysis. As the basic spectral analysis method, PLS has contributed much to spectral analysis works [25,26]. The least squares-support vector machine (LS-SVM) has been demonstrated to be a promising spectral analysis feature after its introduction to the NIR area [27,28,29]. It is known that PLS is quite fast and easy and is simple to operate but lacking accuracy in handling numerous spectral problems, while LS-SVM is strong and robust in most cases but relatively complex and time-consuming. After the selection of characteristic wavelengths, the performance of the LS-SVM model has been greatly improved. Meanwhile, because different data require different kinds of pre-processing progress, we do not have a system-theory for choosing the pre-processing methods for certain kinds of data, and choosing proper pretreatment primarily depends on attempts and experiences, so researchers must devote substantial time and energy to this process. From the point of view of this study, the GF-SNV-LS-SVM model is the optimal model for predicting the number of diastases.

## 4. Materials and Methods

### 4.1. Sample Preparation (Heat Treatment)

A total of 110 original honey samples from three botanical origins were provided by the Institute of Sericulture and Apiculture of Zhejiang University (Hangzhou, Zhejiang, China). The botanical origins were acacia (*n* = 37, one of the main honey sources in summer and the top honey exported from China), linen (*n* = 35, in comparison to acacia and longan), and longan (*n* = 38, the protein content of the single nectar is the highest in China.). Many factors such as floral source, storage, pH, and heating have certain effects on the activity of diastase. Since heating is essential for the commercial process and can change DN, it was adopted in this study as the means to modify the DN of the original honeys through the variation of heating time. Floral source and heating were adopted to acquire different levels of diastase activity for further analysis. Because heating above 40 °C affects the quality and causes protein denaturation and deactivation of several enzymes which are mainly responsible for its functional behavior, heat treatment was applied to the original honeys in order to acquire the gradients of DN [30], and honey thermal treatments were separately carried out in water baths (model of water bath) for 2 h at 40 °C, 2 h at 60 °C, and 2 h, 4 h, 6 h, 8 h, 10 h, and 12 h at 80 °C. To avoid moisture loss during evaporation, samples were heated in some semi-closed test tubes during the heating procedures. Sample settings for the original honey experiment are shown in Table 2.

### 4.2. Reference Method

The diastase activity was measured spectrophotometrically according to the standard method of AOAC (AOAC, 1998 method 958.09). This method was primarily based on the hydrolysis of the starch and iodine test. Starch solution was mixed with certain amounts of honey, and the starch was gradually hydrolyzed due to the existence of diastase in honey. After the addition of the iodine solution, the entire mixture should turn deep blue if there is still much residual starch; the solution turns brown-red colored for partially degraded starch, while it is clear for totally degraded starch. The absorbance of the entire mixture in different reaction times would be measured with a spectrophotometer at 660 nm, and the specified absorbance determined time was needed to calculate DN. As defined, one unit corresponding to the amount of enzyme which will convert 0.01 g of starch to the prescribed end-point in one hour at 40 °C under the conditions of the test. Here, a Jasco Model 7800 UV–Vis spectrophotometer (Japan Spectroscopic Co., Tokyo, Japan) has been utilized.

### 4.3. Spectral Measurement

**Figure 6:**
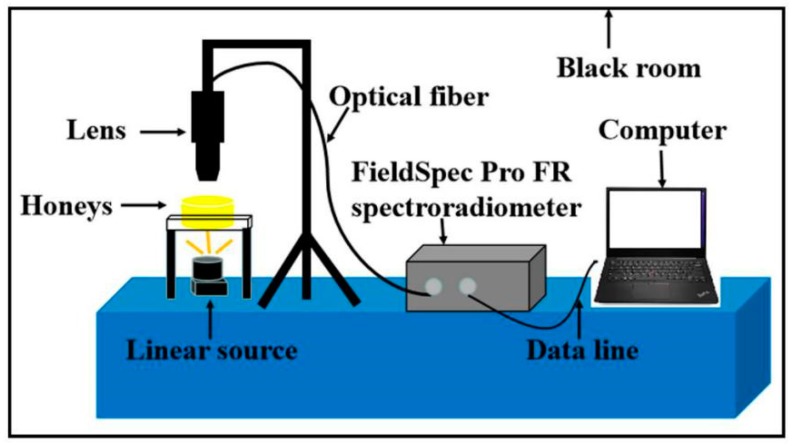
The configuration of the Vis/NIR spectroscopy imaging system. The probe receives the spectral information and transmits it to the spectrometer through the optical fiber. The spectrometer transmits the spectral information to the computer through the data line.

For spectra collection, a handheld FieldSpec Pro FR (325–1075 nm)/A110070 spectroradiometer (Analytical Spectral Devices, Boulder, CO, USA) equipped with a 150 W halogen lamp was used. The field-of-view (FOV) of the spectroradiometer is 25°. The detector on the opposite side captures the light transmitted through honey samples which were alternately placed in a petri dish. Transmission was adopted as the spectral recording mode. Three spectra were recorded for each sample, and the results were ultimately averaged into one value representing the spectrum of the honey. All spectral data were stored in a computer and processed using the RS3 software for Windows (Analytical Spectral Devices, Boulder, CO, USA). A laboratory Vis/NIR spectroscopy imaging system was constructed as shown in Figure 6.

To reduce the interference of instrument noise to the informative spectral data, the first and last 75 wavelength values, the two most noise-rich parts of the entire spectrum, were removed; therefore, the spectral data ranging from 400–1000 nm were finally taken into the analysis. Transmission spectra were transformed into absorbance, which can be related to concentration by Beer’s law, and the absorbance is simply the logarithm of (1/T). The absorbance spectra were then transformed into ASCII format and stored in.txt format in anticipation of the next steps of the analysis. All of the above operations to the data were achieved by using the ASD ViewSpecPro software (Analytical Spectral Devices, Boulder, CO, USA).

### 4.4. Data Analysis

One of the most common ways to analyze a collection of data is to extract the top eigenvectors of a sample covariance matrix that represents the directions of largest variance, often referred to as principal component analysis (PCA) [31]. The aim of the PCA algorithm is to represent the location of the n objects (samples) in a space with reduced dimensionality. In this study, 110 samples (37 acacia, 35 linen, 38 longan) were used in cluster analysis. PCA was conducted based on the spectrum, and the region was conducted in the range of 400–1000 nm.

The successive projections algorithm (SPA) is a useful tool to acquire a small subset of variables with minimum collinearity. It was adapted for characteristic wavelength selection in this research. The variable set with the minimum redundancy could be selected from the spectral information, eliminating collinearity between variables and reducing the number of variables to the minimum [11].

There were many unexpected effects in the process of the experiment, such as light scattering, baseline shifts, and random noise. To eliminate these unexpected effects, spectral data are subjected to pretreatment. Different pre-processing methods, including Savitzky-Golay (SG) smoothing, Savitzky-Golay smoothing-standard normal variate (SG-SNV), Gaussian filter smoothing-standard normal variate (GF-SNV), multiplicative scatter correction (MSC), and Savitzky-Golay second derivative (S-G DER) were compared in this study. By comparing different pretreatment effects, the best pretreatment method is selected.

Calibration methods, including partial least squares (PLS) and least squares-support vector machine (LV-SVM) models, were used; because the non-linear factors existed in the spectra, PLS was destined to fail to some extent, but LS-SVM was hoped to fulfill the prediction task. LS-SVM, which aims to simplify the computational process and extend the framework of standard SVM, was proposed by Suykens et al. in 1999 [32]. LS-SVM finds the solution by solving a set of linear equations instead of convex quadratic programming for classical SVMs, and it is closely linked with Gaussian processes and regularization networks but also additionally emphasizes and exploits primal-dual interpretations from optimization theory, making it useful in either linear and nonlinear classifications or function estimations.

All of the calculations with respect to LV-SVM in this paper were executed in MATLAB v7.0 (The Math Works, Natick, MA, USA), and a free LS-SVM toolbox for MATLAB (LS-SVM v 1.5; Johan Suykens, Leuven, Belgium) was used as the software support. Furthermore, all of the pretreatments and PLS models were implemented by Unscrambler v9.6 (CAMO PROCESS AS, Oslo, Norway). The following two parameters, the determination coefficient (*R*^2^) and root mean square error of prediction (RMSEP), were used to estimate the performance of the calibration model.

(1) $$R^{2}=\left[1-\frac{\sum_{i=1}^{n}\left(y_{i}-y_{i}^{\prime }\right)}{\sum_{i-1}^{n}\left(y_{i}-y_{a}\right)}\right]\times 100\%$$

(2) $$RMSEP=\sqrt{\frac{1}{n}\sum_{i=1}^{n}\left(y_{i}-y^{\prime }{}_{i}\right)}$$

## 5. Conclusions

The kinetic mechanism of the temperature effect on diastase activity was studied. Diastase numbers in dissimilar floral sources were different, and the sensitivity to temperature was also dissimilar. The sensitivity of acacia and longan honeys to temperature and diastase activity was much higher than that of linen. The diastase activity decreased with the increase of temperature and heating time. To compare the effects of different processing temperatures on diastase activity, it is recommended that the honey production and processing temperature be 60 °C; SPA selected 6 characteristics spectra in the visible region and obtained good prediction results. Vis/NIR spectroscopy can detect the vibration and stretching of O-H (6ν), O-H (4ν), and C-H (4ν) to predict DN in honeys. Different pre-processing methods were attempted in both of the PLS and LS-SVM models, and the results indicated that the combination of GF-SNV and LS-SVM was quite successful in acquiring a preferable prediction result of diastase activity using the spectra ranging from 400–1000 nm, which was improved after the selection of characteristic wavelengths. Revealing the characteristic band assignment of the near-infrared spectroscopy detection of diastase activity in honey processing reflects the spectrum detection mechanism of diastase activity and has great significance for the development of portable and sixpenny on-line detection equipment. All in all, rapid and non-destructive testing of diastase activity during honey processing has great value in improving and maintaining the nutritional activity in honey processing, and the study finally demonstrates that it is possible for the NIR technique to attain fairly useful and accurate results with respect to the rapid evaluation of diastase activity of honeys.

## Figures and Tables

**Table 1:** Results of models with different spectral pre-treatments.

Regression Algorithm	Pre-Treatment	Calibration		Prediction	
		*R* ^2^	RMSE	*R* ^2^	RMSE
PLS	ORIG	0.6086	0.4149	0.4636	0.4642
	SG	0.6093	0.4145	0.4674	0.4625
	SG-SNV	0.7049	0.3602	0.6753	0.3612
	GF-SNV	0.7057	0.3597	0.6720	0.3630
	MSC	0.7452	0.3348	0.5853	0.4082
LS-SVM	ORIG	0.9772	0.1339	0.6017	0.4000
	SG	0.9808	0.1239	0.5350	0.4322
	SG-SNV	0.9988	0.0321	0.8857	0.2142
	GF-SNV	0.9785	0.1301	0.8872	0.2129
	MSC	0.9966	0.0533	0.8269	0.2637

**Table 2:** Sample settings for the original honey experiment.

	Treatment	40 °C	60 °C	80 °C	80 °C	80 °C	80 °C	80 °C	80 °C
Number		2–4 h	2–4 h	2 h	4 h	6 h	8 h	10 h	12 h
Acacia		5	5	5	5	5	5	2	5
Linen		5	5	5	5	5	4	3	3
Longan		5	5	5	5	5	5	4	4

## Supplementary Materials

The following are available online at https://www.mdpi.com/1420-3049/24/7/1244/s1, Table S1: Samples spectral data, Table S2: The diastase activity for each sample.
